# Mature Solid Teratoma of the Retroperitoneum Presenting as Sigmoid Colon Polyp with Lower Gastrointestinal Bleeding: A Case Report

**DOI:** 10.4314/ejhs.v32i5.19

**Published:** 2022-09

**Authors:** Nnaemeka Thaddeus Onyishi, Aiah Lebbie, Alie Amin Sesay, Babatunde Moses Duduyemi

**Affiliations:** 1 Anatomic pathology Department, University of Sierra Leone Teaching Hospital Complex Freetown Sierra Leone; 2 Paediatric Surgery Unit University of Sierra Leone Teaching Hospital Complex Freetown Sierra Leone

**Keywords:** sigmoid teratoma, sigmoid polyp, gastrointestinal teratoma

## Abstract

**Background:**

Mature colonic teratomas are rare tumors and no case, to the best of our knowledge, has been reported from the African continent. In addition, some pedunculated teratomas in the colon have been treated by endoscopic polypectomy and classified as primary teratoma of the colon. We report a case of a distinct intra sigmoid pedunculated teratoma originating from the retroperitoneum of a 4-year-old African girl and we highlight the potential for misclassification of primary origin of endoscopically removed polypoid teratomas in the colon.

**Case Presentation:**

A 4 year-old black African female child who presented with abdominal pain and hematochezia. On clinical assessment, she was found to be anaemic and to have a sigmoid colon mass. At surgery, there was a mobile mass within the lumen of sigmoid colon and the mass was fixed to the retroperitoneum by a stalk of tissue. Pathologist's review of the resected sigmoid segment showed a pedunculated intra-sigmoid mass with the stalk traversing the wall of the colon. The mass was histologically proven a mature solid teratoma.

**Conclusions:**

This, to the best of our knowledge, is the first report of intra sigmoid teratoma from the African continent. It highlights the potential for misclassification of endoscopically resected colonic teratomas.

## Introduction

Teratomas are germ cell tumours that can occur in gonadal and extragonadal sites. The most common sites of teratoma in children, in descending order of frequency, are sacrococcygeal, mediastinal, gonadal and retroperitoneal sites (1). Also reported, though less commonly, are teratomas of the intracranial, cardiac and gastrointestinal sites (2,3). Along the gastrointestinal tract, the stomach has been found the most common location of teratomas (1,2). Extremely rare are teratomas of the sigmoid colon of which there are few featured case reports (4). A thorough literature search returned no reported case in Africa. A number of reported intraluminal colonic teratoma had intricate relations with pericolonic tissues and some were demonstrated as fistulation of ovarian teratoma into the colon(5,6). We report a rare case of mature solid teratoma arising in the retroperitoneum in a 4 year old girl and presenting as sigmoid colon polypoid mass with lower gastrointestinal bleeding thus demonstrating that a neatly pedunculated intra colonic teratoma can be of extra-intestinal origin.

## Case Report

A 4 year-old black African female child was referred from a district hospital with complaint of left lower quadrant abdominal pain and hematochezia of 8 weeks. This was associated with anorexia but there was no weight loss or vomiting. She had been transfused with one unit of blood at the referring hospital. On examination, her pulse rate was 120 beats per minute. She was anaemic with haemoglobin concentration of 7.9g/dl.

There was a mobile intraabdominal mass measuring 8 x 8 cm located in the left side of the abdomen. Digital rectal examination revealed normal anal anatomy and soft stool in the rectum. Abdominal ultrasound showed an abdominal mass probably arising from the sigmoid colon. Patient could not afford an abdominal CT scan and was then worked-up for exploratory laparotomy. At surgery, there was a mass within the lumen of proximal sigmoid colon with a stalk extending into the retroperitoneum. Intestinal resection and anastomosis was done, patient had an uneventful post-operative recovery and was discharged home on postop day 5.

**Figure 1:**
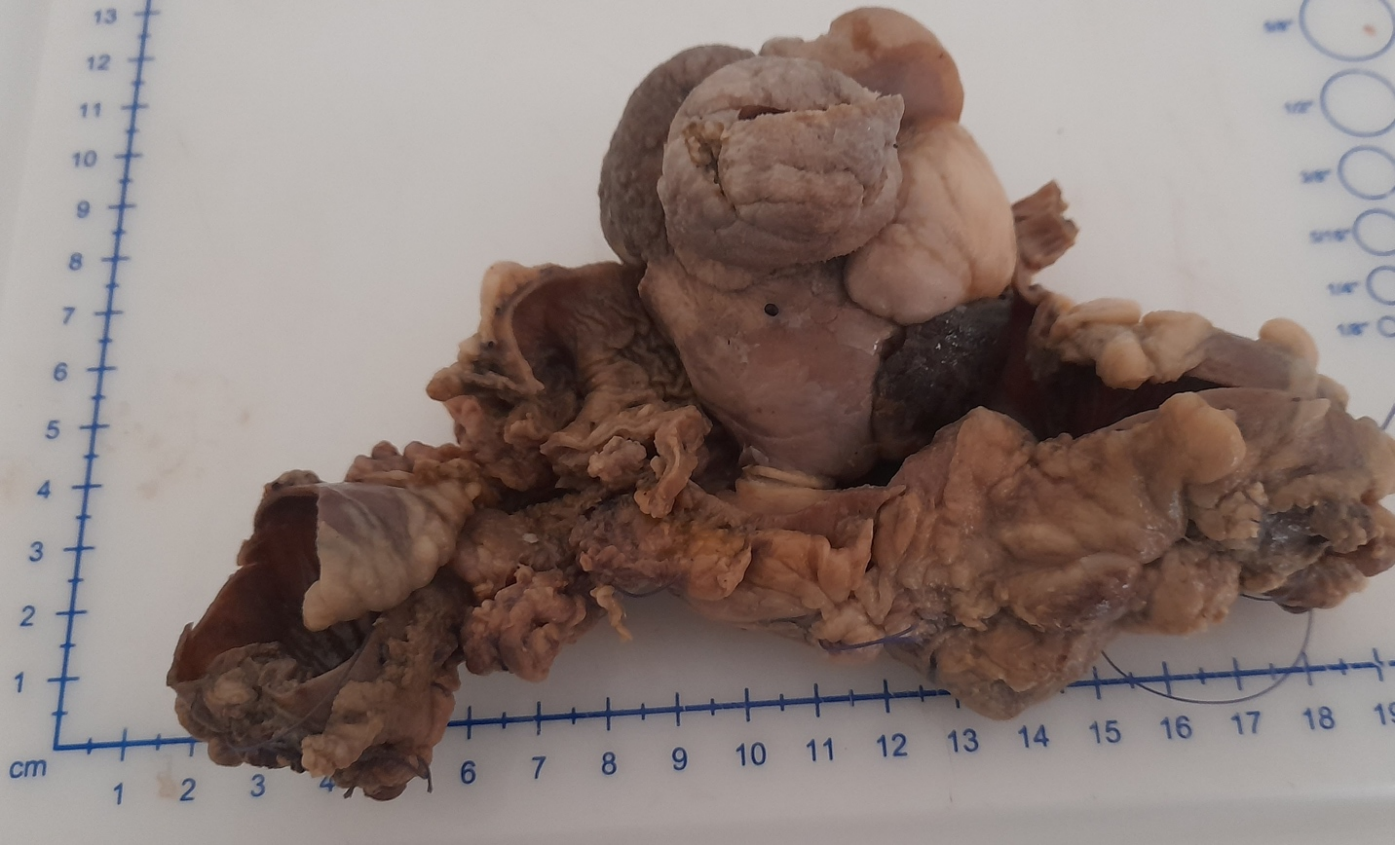
Formalin-fixed sigmoid colon resection segment showing an intraluminal polypoid teratoma

Macroscopic analysis of the resected intestine showed a segment measuring 17cm in length. On opening, there was a lobulated pedunculated polypoid mass protruding into the lumen. The mass measured 7 x 6 x 5 cm and its cut surface was entirely solid. The stalk was slender, measured 0.6cm in diameter and traversed the tunics of the colon to the exterior (Figure 1).

**Figure 2:**
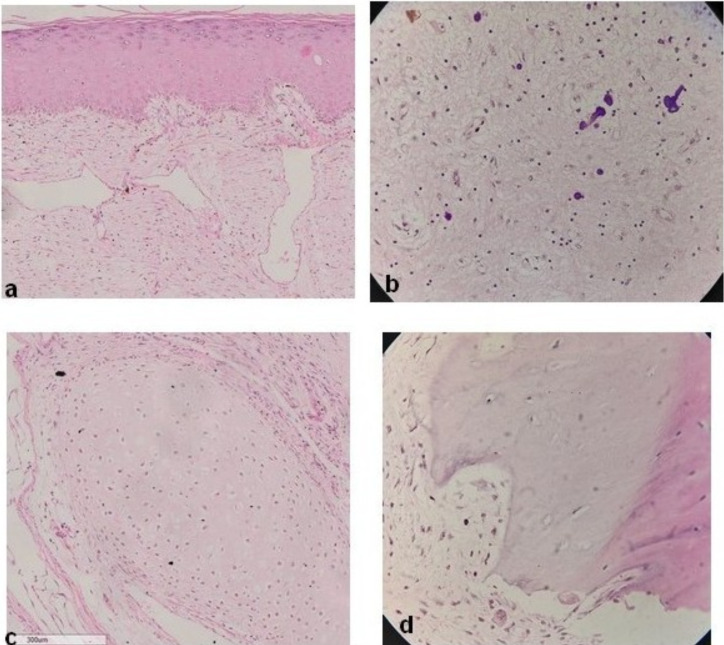
Photomicrograph of some tissue types present in mature solid teratoma presenting as polypoid mass in the sigmoid colon (H & E stain; X40 objective): (a) Keratinized skin(adnexal structures were present in other fields),(b) Mature glial tissue with calcific foci, (c) Cartilage, (d) Bone

Histologic sections showed tissues derived from embryonic germ layers. The ectodermal derivatives were represented by cornified skin with skin appendages and mature brain tissue. Mesodermal tissues present included lobules of adipocytes, fibrous tissue, cartilage and bone. Some other foci showed glandular structures (Figure 2). Histology of the connecting stalk showed bland fibrovascular connective tissue. All tissues present were mature, prompting a diagnosis of mature solid teratoma.

## Discussion

Though documented in literature, teratomas of the gastrointestinal tracts are rare lesions(2,4). Most of the teratomas diagnosed in the gastrointestinal tract have been of the stomach. To date, up to 150 cases of gastric teratomas have been reported occurring mostly in infant males (1,2). In the large bowel, the rectum presents most teratomas with about 65 cases reported till date(7). Sigmoid teratomas are much less frequent with just about 7 cases documented in literature (4,6,8). Almost all cases of rectal teratomas and other colonic teratomas have occurred in females (7). To the best of our knowledge, no case of gastrointestinal teratoma has been reported from the African continent. The present case is a 4 -year-old child who presented with an intraluminal sigmoid teratoma. Clinically, the tumour caused hematochezia and anaemia. Intra-operatively, there was an intra-sigmoid colon mass and the sigmoid was found fixed to the retroperitoneum by a pedicle. Gross examination of the resected colon showed an intraluminal pedunculated mass with the pedicle traversing the wall of the intestine to the exterior.

When faced with teratoma involving the large intestine, one requisite diagnostic determination to be made is the origin of the tumour in question; whether it is primarily of the colon or a secondary involvement of the intestine by a gonadal or an extragonadal tumour. There are a number of reports of benign ovarian teratomas fistulating into large intestine and it is recognized that some luminal teratomas of the colon are extra intestinal in origin (5).

Our case was a pedunculated intraluminal mass. Most teratomas of the colon have been found to be polypoid or pedunculated and Palombini et al did hypothesize that pedunculation might arise from peristaltic traction (8). Pedunculation, if present enables endoscopic resection of a colonic teratoma. Most of the endoscopically resected colonic teratomas were classified as primary teratomas of the colon. Our case would have been similarly classified if it was treated by endoscopic polypectomy, but colectomy enabled us determine the retroperitoneal and extra intestinal origin of what was otherwise, a distinct colonic polyp.

In conclusion, this case, to the best of our knowledge, is the first report of intra sigmoid teratoma from African continent. More crucially, it highlights the potential for misclassification of endoscopically resected colonic teratomas.
